# Excellent Bipolar Resistive Switching Characteristics of Bi_4_Ti_3_O_12_ Thin Films Prepared via Sol-Gel Process

**DOI:** 10.3390/nano11102705

**Published:** 2021-10-14

**Authors:** He-Chun Zhou, Yan-Ping Jiang, Xin-Gui Tang, Qiu-Xiang Liu, Wen-Hua Li, Zhen-Hua Tang

**Affiliations:** School of Physics and Optoelectric Engineering, Guangdong University of Technology, Guangzhou Higher Education Mega Centre, Guangzhou 510006, China; hechunzz@163.com (H.-C.Z.); xgtang@gdut.edu.cn (X.-G.T.); liuqx@gdut.edu.cn (Q.-X.L.); liwenhuat@gdut.edu.cn (W.-H.L.); tangzh@gdut.edu.cn (Z.-H.T.)

**Keywords:** Bi_4_Ti_3_O_12_, resistive switching, oxygen vacancies, conductive filaments, thin film

## Abstract

Herein, Bi_4_Ti_3_O_12_ (BIT) ferroelectric thin films were fabricated into Au/BIT/LaNiO_3_/Si structures to demonstrate their memristor properties. Repeatable and stable bipolar resistive switching (RS) characteristics of the device are first reported in this work. The switching ratio of the device annealed in air reached approximately 10^2^ at 0.1 and −0.1 V. The RS performance was not significantly degraded after 100 consecutive cycles of testing. We also explored the factors affecting the RS behavior of the device. By investigating the RS characteristics of the devices annealed in O_2,_ and in combination with XPS analysis, we found that the RS properties were closely related to the presence of oxygen vacancies. The devices annealed in air exhibited a markedly improved RS effect over those annealed in O_2_. According to the slope fitting, the conduction mechanism of the device was the ohmic conduction and space charge limited current (SCLC). This study is the first to successfully apply BIT ferroelectric films to the RS layers of memristors. Additionally, a theory of conductive filaments is proposed to adequately explain the relationship between RS behavior and oxygen vacancies, providing meaningful inspiration for designing high-quality resistive random access memory devices.

## 1. Introduction

Information storage technology is one of the most rapidly developing areas in the field of integrated circuits. Memory is the most critical core of information storage technology [1,2]. As traditional memristors can no longer satisfy the requirements of improving information storage and programming speed, developing a new generation of memristors has gained scholarly interest in recent years [3,4]. Numerous studies have shown that a non-volatile memory has a significant advantage over a volatile memory in the read-write operation of a storage system due to its capability to maintain information in case of power failure [5,6]. To date, several competitive non-volatile memories have been developed, including but not limited to phase change, magnetic, ferroelectric, and resistive random-access memory (RRAM) [7,8,9]. Given its excellent scalability, simple structure, high-endurance cycles, fast operation, and high-density data storage, RRAM is considered an ideal candidate to meet the demands for the promising non-volatile memory of the future. Regarding its application in information capacity, however, there are a variety of issues that need to be addressed, such as retention characteristics, fatigue, and transmission mechanisms [10,11]. In general, one of the most significant advantages of a typical RRAM lies in its simple metal-insulator-metal (MIM) device structure, in which resistive switching (RS) layers are sandwiched between two metal electrodes. When the bias polarity or voltage amplitude is stimulated, RRAM can electrically change its resistance state between a high-resistance state (HRS) and low-resistance state (LRS) to complete the storage of information [12,13]. When the “SET” to LRS state occurs at one bias polarity and the “RESET” to HRS state on the opposite bias polarity, the RS can be considered a bipolar RS. Conversely, if the switching procedure only requires the amplitude of the same polarity to obtain the SET and RESET, the RS is taken into account as a unipolar RS [11,14]. To date, the RS characteristic of an insulator layer has been explored in different materials, including transition metal oxides (TiO_2_, ZnO, NiO, Ta_2_O_5_, HfO_2_, and Al_2_O_3_ [15,16,17,18,19,20]), perovskite oxides (SrTiO_3_, BaTiO_3_ [21,22]), etc. On the other hand, RRAM devices based on a MIM sandwich structure cannot be separated from thin-film synthesis technology. In recent years, breakthroughs have been made in film synthesis technology. Many physical and chemical methods have been used to synthesize films, such as atomic layer deposition (ALD) [23], sol-gel method [24], and metalorganic chemical vapor deposition (MOCVD) [25]. Among them, the sol-gel method has been widely used due to its advantageous large-area uniformity, simple equipment, and relatively low processing temperature.

Recently, Bi_4_Ti_3_O_12_ (BIT) thin film, a bismuth layer-structured ferroelectric, was reported to be a promising material replacement of conventional lead zirconate titanate, used for various electronic components due to its relatively low crystallization temperature, excellent fatigue endurance, as well as for being environmentally friendly [26]. Additionally, BIT was studied as a promising photocatalyst with a narrow bandgap below 3 eV, highlighting its outstanding photoelectric response performance [27]. However, BIT thin films with both ferroelectric and RS properties have not been reported in studies. LaNiO_3_(LNO) is a kind of pseudocubic perovskite metal oxide with stable chemical properties and good electrical conductivity. The lattice mismatch with many ferroelectric thin films is small, which not only affects the crystal orientation of thin films, but also improves the electrical properties of ferroelectric thin films [28]. In recent years, LNO has mainly been used to replace Pt/Ti and other metals as electrode materials of ferroelectric thin films, such as bismuth titanate and lead zirconate titanate, and has been applied in microelectronics and electronics [29,30]. In this study, BIT films were deposited on LNO/p-Si substrate through the sol-gel process. We explored the RS characteristics, durability, and conductivity of BIT-based memory devices.

## 2. Materials and Methods

Here, BIT thin films grown on LNO/p-Si substrates were prepared via the sol-gel process. All chemical reagents used in the experiment were of analytical grade. A LaNiO_3_ precursor solution was prepared using lanthanum nitrate La(NO_3_)_3_·6H_2_O (Macklin, Shanghai, China) and nickel acetate Ni(OCOCH_3_)_2_·4H_2_O (Macklin, Shanghai, China) as raw materials. The solvent used was glacial acetic acid (Aladdin, Shanghai, China). La(NO_3_)_3_·6H_2_O and Ni(OCOCH_3_)_2_·4H_2_O were dissolved in glacial acetic acid according to a certain stoichiometric ratio and stirred until the solute was dissolved. The concentration of the solution was adjusted to 0.3 mol/L. First, the p-Si substrate was placed on a homogenizer (Jinyi, China) and the LNO solution was then dropped onto the p-Si substrate, and rotated at 3000 rpm for 40 s. Finally, the sample was annealed in rapid thermal annealing equipment (Kejing, Hefei, China) at 650 °C for 10 min. Bismuth nitrate pentahydrate Bi(NO_3_)_3_·5H_2_O (Kermel, Tianjin, China) and tetra-n-butyl titanate Ti(C_4_H_9_O)_4_ (Macklin, Shanghai, China) were selected as raw materials to prepare the precursor solution of Bi_4_Ti_3_O_12_. The solvent used was acetic acid with 2-methoxy ethanol (C_3_H_8_O_2_) (Aladdin, Shanghai, China). An excess of 10 mol % bismuth nitrate pentahydrate was added to compensate for the volatile Bi. Firstly, Bi(NO_3_)_3_·5H_2_O was dissolved in a mixed solution consisting of C_3_H_8_O_2_ and acetic acid at a volume ratio of 1:1. Then, Ti(C_4_H_9_O)_4_ was dissolved in acetylacetone (C_5_H_8_O_2_) (Macklin, Shanghai, China) at room temperature. Next, the two solutions were mixed and stirred at room temperature for 3 h until fully dissolved. Afterward, a 2-methoxy ethanol solvent was added to adjust the concentration of the solution to 0.05 mol/L. No additive was mixed with the final solution after an appropriate duration, which was ready for fabrication. Then, the precursor solution was filtered using a filter and used for spin-coating. The precursor solution was dropped in a clean LNO/p-Si substrate using a syringe. Thereafter, the LNO/p-Si substrate was placed on the platform of the rotary coating machine for spin-coating at 2000 rpm for 20 s, and then at 4000 rpm for 30 s. The as-coated films were set on a plate at 300 °C for 15 min to promote thermal decomposition. The above-mentioned process was repeated three times to achieve a film thickness of 120 nm. Afterward, the devices were annealed in 700 °C air for 20 min by using rapid thermal annealing equipment (Kejing, Hefei, China). After annealing, the Au dots with diameters of 0.5 mm were prepared on the top layer of the film of the top electrode by using an ultra-high vacuum chamber (Saintins, Beijing, China).

The grazing incidence X-ray diffraction (Bruker, Bremen, Germany) was employed to analyze the crystal structures of the BIT and LNO-buffered film. The current-voltage (I-V) characteristics of the device were analyzed using a Keithley 2400 (Solon, OH, USA) semiconductor parameter analyzer. The surface and cross-sectional image of the sample were captured using scanning electron microscopy (Hitach, Tokyo, Japan). The O 1s spectrum of the BIT films was measured using X-ray photoelectron spectroscopy (Thermo Fisher, Manchester, England). Moreover, the ferroelectric properties of the BIT film were investigated by a ferroelectric test system (Radiant, Redmond, WA, USA).

## 3. Results

### 3.1. Structure Analysis

**Figure 1:**
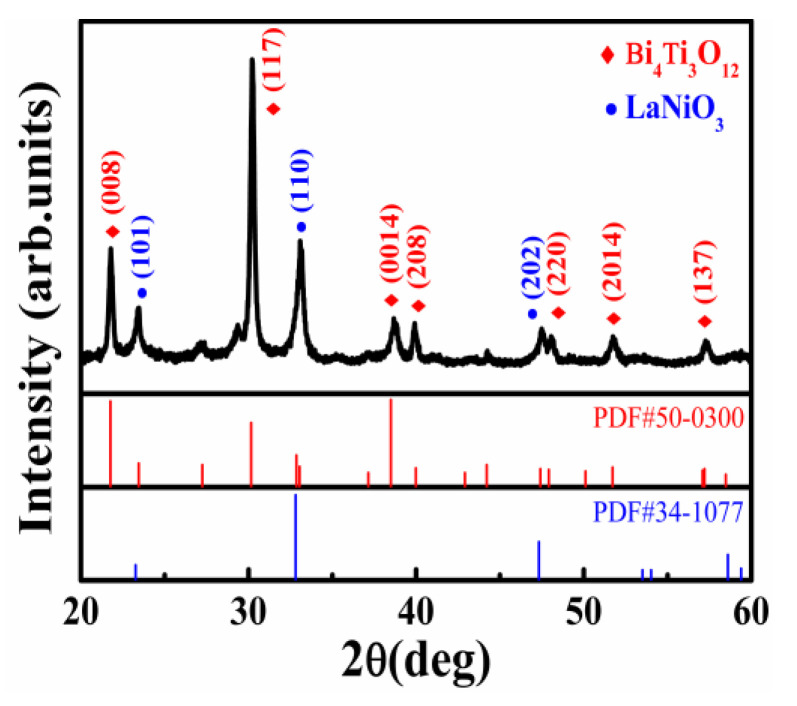
XRD patterns of the Au/BIT/LNO/p-Si sample.

**Figure 2:**
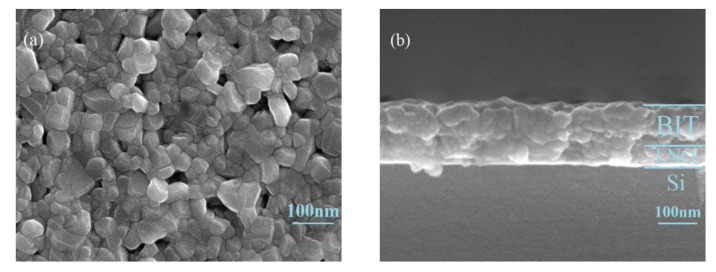
SEM micrographs of the BIT films prepared on LNO/p-Si substrate annealed at 700 °C for 20 min. (**a**) Topography image, and (**b**) cross-sectional view.

The BIT films were successfully deposited on p-Si substrates with LNO-buttered films via the sol-gel method. Figure 1 exhibits the XRD patterns of BIT films grown on the LNO/p-Si substrate. The diffraction peaks of the LNO-buffered films are also displayed in Figure 1 for comparison. The peaks marked in red are consistent with the diffraction data of the standard card PDF#50-0300, indicating the BIT film is polycrystalline and has a single phase of the bismuth-layered perovskite structure. The BIT film shows a preferred orientation of (117), which may be due to the effect of the high annealing temperature. The remnant peaks at 23.26°, 32.81°, and 47.33° belong to the (101), (110), and (202) lattice planes of hexagonal LNO, respectively, corresponding to the blue standard card PDF#34-1077. Furthermore, there were no intermediate or additional peaks apart from BIT films and LNO-buffered films. Figure 2a shows the surface SEM photographs of the samples. The BIT films are well-crystallized. The crystal grains are clearly visible, and their diameters range from about 30 to 70 nm. Figure 2b shows the cross-sectional view of the sample. The thickness of the BIT films and LNO-buffered films are approximately 120 and 60 nm, respectively. In addition, XPS was also used to study the chemical composition and elemental oxidation states of BIT-thin films. The results are shown in Figure S1 of the Supplementary Materials. The results show that no other impurities were introduced into the sample during the preparation process.

### 3.2. Electrical Performance

**Figure 3:**
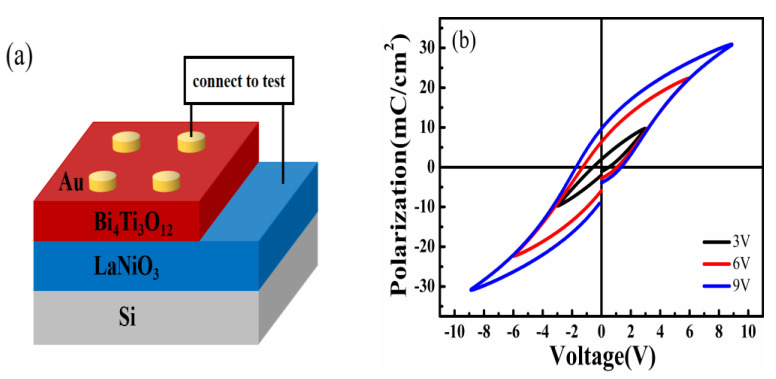

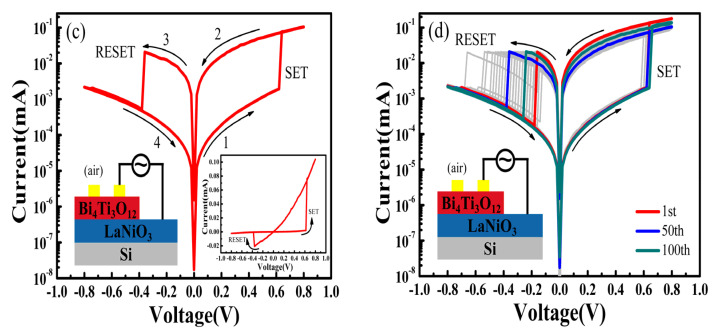
(**a**) Schematic drawing of the Au/BIT/LNO/Si device. (**b**) P−V loops of the device at 1 KHz by increasing the voltage sweep range. (**c**) Semilogarithmic I−V curve. The inset shows the I−V curve of the device. (**d**) Semilogarithmic I−V curve of the device under 100 voltage sweep cycles.

To test the electrical properties of the Au/BIT/LNO/Si device, the ferroelectric and I-V performances were investigated according to the schematic diagram of the device shown in Figure 3a. Figure 3b shows the room-temperature polarization-voltage (P-V) hysteresis loops of the device measured at 1 KHz. It is shown that the ferroelectric hysteresis loop begins to appear when the bias voltage is greater than 3 V. It shows symmetric hysteretic loops. When the applied bias voltage reached 9 V, the maximum polarizability (2Pmax) and residual polarizability (2Pr) were 61.86 and 19.38 mC/cm^2^, respectively, indicating that the BIT film exhibits good ferroelectricity. Furthermore, the coercive voltages of the films were about −1.7 V for negative bias and +1.3 V for positive bias. Due to the differences between the Au/BIT and BIT/LNO interfaces, a built-in electric field forms in the BIT film and results in the asymmetry of coercive voltages, which has been widely observed in asymmetrical metal ferroelectric metal (MFM) devices [31].

To investigate the RS characteristics of the Au/BIT/LNO/Si device, I-V curves were measured at room temperature according to the schematic illustration depicted in Figure 3a. The inset in Figure 3c displays the I-V characteristics of the device. The arrows indicate the voltage-sweeping directions. The order of voltage scanning is 0 V→0.8 V→0 V→−0.8 V→0 V. We observed that the device has a diode-like rectification effect and traditional resistance-switching characteristics. To analyze the RS characteristics of the device, a semilogarithmic plot of the I–V measurements is shown in Figure 3c. The device was initially at the HRS. In the “1” path, with the increase in the positive bias, a SET occurred at about 0.6 V, where the current of the device was markedly elevated, and the HRS was simultaneously converted to the LRS. Afterward, with the voltage scanned in “2” and “3”, the LRS was maintained until the voltage reached about −0.4 V. Then, an adverse process formed, in which RESE” took place at around −0.4 V, and the LRS was simultaneously triggered to the HRS. Finally, the HRS followed in its initial state for the next uninterrupted sweeping cycle. To further assess the durability and stability of the RS characteristics of the devices, the RS characteristics with 100 voltage sweeping cycles are illustrated in Figure 3d. The semi-logarithmic I-V curves measured after 100 cycles were found to be similar to those measured in the first cycle. We observed that the Au/BIT/LNO/Si memory device possesses excellent forming-free bipolar, non-volatile RS characteristics.

**Figure 4:**
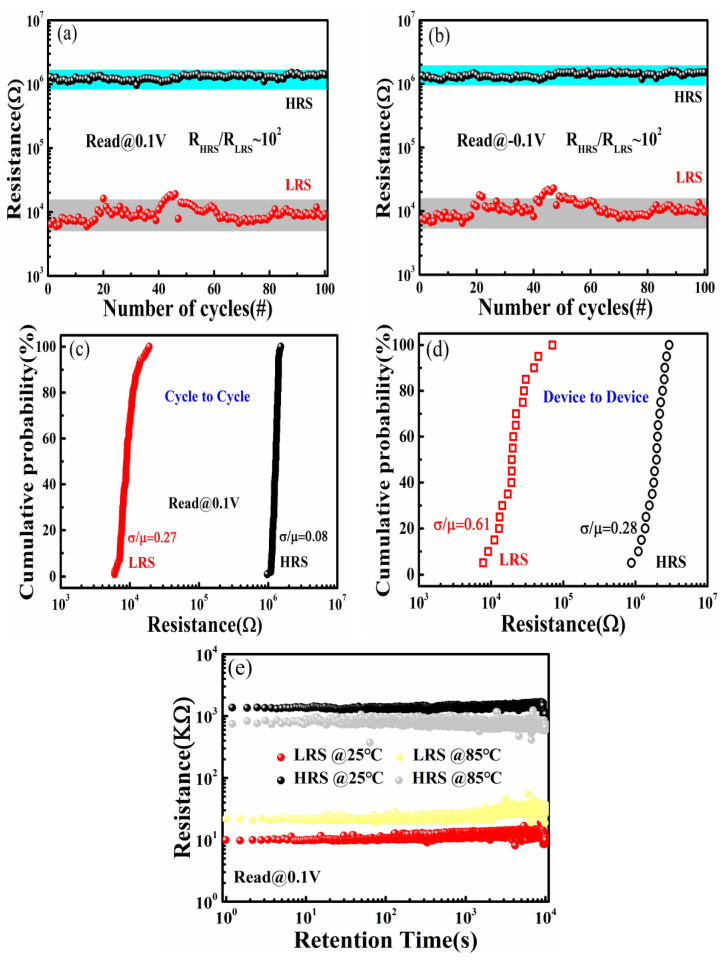
Endurance and retention test results of the device. The HRS and LRS read at (**a**) 0.1 V and (**b**) −0.1 V for 100 cycles. The cycle-to-cycle distribution of HRS and LRS for 100 cycles in (**c**), device-to-device distribution of HRS and LRS for 20 devices in (**d**), the retention characteristics of (**e**), and HRS and LRS at room temperature (25 °C) and 85 °C.

The resistance state values of the HRS and LRS measured at 0.1 and −0.1 V for 100 consecutive cycles are displayed in Figure 4a,b, respectively. We found that a larger ratio of R_HRS_/R_LRS_ can be obtained at smaller voltages of −0.1 V and 0.1 V. It is noteworthy that the overall HRS and LRS tend to be stable. Moreover, the ratio of RHRS/RLRS of the device could reach 10^2^ and did not remarkably degrade after 100 cycles, demonstrating that the device has good durability and stable, repeatable read/write characteristics. To explore the RS uniformity of devices, the cycle-to-cycle and device-to-device distributions were investigated and are shown in Figure 4c,d, respectively. The relative standard deviation (σ/μ) was used to indicate the degree of variation. The cumulative probability of cycle-to-cycle distribution of the HRS and LRS was extracted from 100 consecutive switching cycles within a device, as shown in Figure 4c. The devices manifest a stable distribution for the on/off properties. The cumulative probability of the device-to-device distribution in the HRS and LRS was obtained from 20 randomly selected devices, as shown in Figure 4d. The results show a low device-to-device variability, indicating the device has good reliability and repeatability. To confirm its potential in memory storage applications, we investigated the data-retention characteristics of the device at room temperature (25 °C) and 85 °C. As shown in Figure 4e, the two resistance states of HRS and LRS are stable over 10^4^ s at 25 and 85 °C under 0.1 V, without significant degradation. The excellent data-retention performance of the device demonstrates the potential for non-volatile memory applications. Table 1 compares key performance aspects of the Au/BIT/LNO/Si device with other recently reported devices.

**Figure 5:**
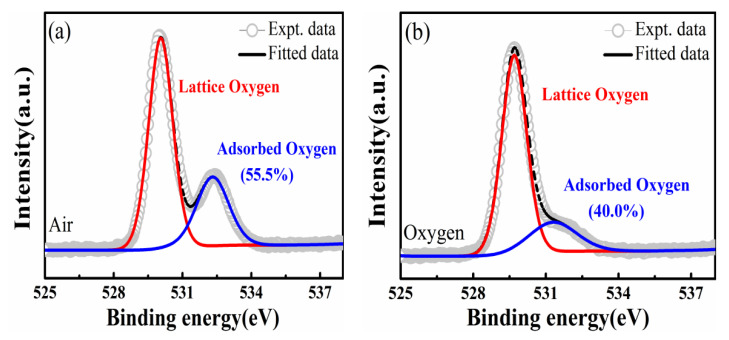
XPS high-resolution spectra of O 1s of the BIT film annealed in (**a**) air and (**b**) oxygen atmospheres.

**Figure 6:**
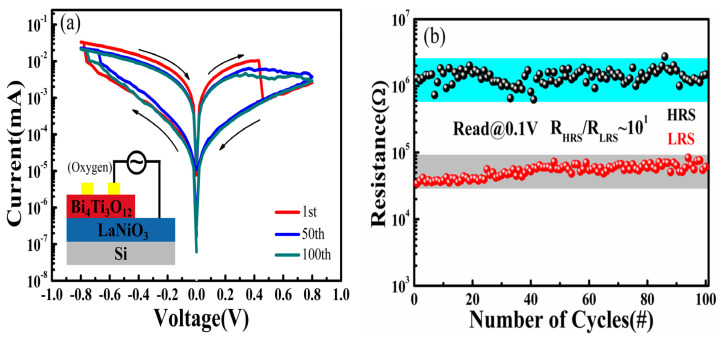
(**a**) Semilogarithmic I−V curve of the device under 100 voltage sweep cycles. (**b**) Resistance of HRS and LRS read at a bias of +0.1 V.

To analyze the influence of oxygen vacancies in the RS properties, we remanufactured a sample annealed in O_2_, keeping the other experimental processes the same as that of samples annealed in air. Moreover, the XPS test was conducted for both of the devices. To date, many studies have shown that XPS results can confirm the existence of oxygen vacancies [36,42]. As shown in Figure 5a, the O 1s spectrum of the devices annealed in air was fitted by two Gaussian peaks at 529.5 and 531.5 eV. The Gaussian peak with a lower binding energy at 529.5 eV was indexed to the crystal lattice oxygen, and the other with a higher binding energy at 531.5 eV arose from adsorbed oxygen, which could be associated with the oxygen vacancies in the BIT film [43,44]. In Figure 5b, the O 1s narrow sweep spectrum of the device annealed in O_2_ also deconvoluted into two clearly separated Gaussian peaks at 529.8 eV and 531.6 eV, defined as crystal lattice oxygen and adsorbed oxygen, respectively. The ratio of the area of adsorbed oxygen to the total peak area can be defined as the content of oxygen vacancies [44,45]. The results show that the content of oxygen vacancies of the device annealed in air is significantly higher than that of the device annealed in O_2_, which is 55.5% and 40.0%, respectively. In addition, the RS properties and the high/low resistance state values of the device annealed in O_2_ are depicted in Figure 6a,b, respectively. The results show the ratio of R_HRS_/R_LRS_ of the device annealed in O_2_ was much smaller than the device annealed in air, by about 10^1^. This demonstrates that the oxygen vacancies in BIT film are core to its RS characteristics. The results show that the existence of oxygen vacancies plays a crucial role in determining the RS properties of the Au/BIT/LNO/Si device. Higher concentrations of oxygen vacancies in these materials can effectively enhance resistance switching properties, as also found in previous work [46].

### 3.3. Mechanism Discussion

**Figure 7:**
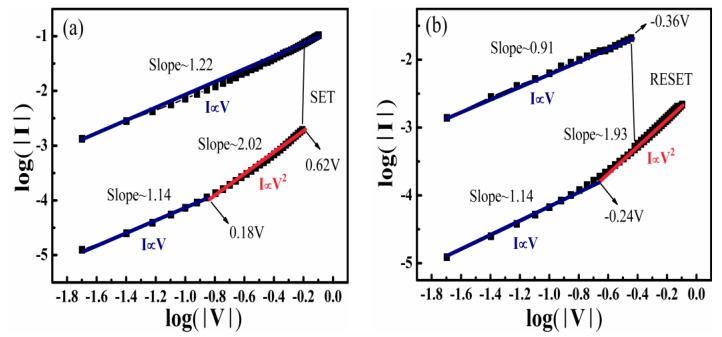
(**a**) Log(I)−log(V) plots of the device under positive voltage and (**b**) under negative voltage.

To interpret the conduction mechanism of the RS properties, the I-V double-logarithmic curves of the device annealed in air were selected to fit and analyze the carrier transmission. The positive and negative voltage regions are shown in Figure 7a,b, respectively. For the LRS, the slope obtained by the fitting curves for both positive and negative sweeps is roughly equal to one, demonstrating that the conduction mechanism is regulated by Ohm’s law. For the HRS, the slope of the fitting curves in the low-voltage region (0 ≤V≤ 0.18 V for the positive voltage region, and −0.24 ≤V≤ 0 V for the negative voltage region) is close to one, indicating that the initial HRS state follows Ohm’s law. The I-V characteristics of Ohmic conduction can be expressed as [47]:

(1) $$J_{Ohm}=qn_{0}\mu (V/d)$$

where *J_Ohm_* is the current density, q is the elementary charge, *n*_0_ is the free carrier density, μ is the carrier mobility, d is the film thickness, and *V* is the applied bias.

However, in the high-voltage region in the HRS (0.18 ≤V≤ 0.62 V for the positive voltage region, and −0.24 ≤V≤ −0.36 V for the negative voltage region), the slope is approximately equal to two (the slope for the positive voltage region is equal to 2.02, and the slope for the negative voltage region is equal to 1.93), which was subjected to the space-charge-limited conduction (SCLS; Child’s law). It was indicated that the threshold voltage from the Ohmic conduction area to the Child’s square area corresponded to the transformation from the trap-unfilled region to the trap-filled region of the SCLC mechanism [48,49]. The conduction current density of SCLC can be expressed as follows [50],

(2) $$J_{SCLC}=(9/8)\mu \varepsilon (V^{2}/d^{3})$$

where *μ* is the electron mobility, ε is the permittivity of the film, d is the thickness of the film, and V is the applied voltage. In SCLC theory, an electron defect trap is treated as an oxygen vacancy. With a rise in voltage corresponding to the increase in injected carriers, oxygen vacancies are filled with electrons, and the current passing through the film increases. At the time when defects composed of oxygen vacancies brim with injected electrons, the current will noticeably increase, representing a switch from the HRS to LRS [51]. In addition, both the positive and negative voltage regions of the conduction mechanism of the LRS were governed by Ohm’s law, indicating the conductive filaments formed in the BIT films [52]. The most prominent feature of the filament principle is the sudden increase or decrease in the current during the SET and RESET steps. Thus, this further indicates that the conductive filaments control the resistance switching characteristics of the device.

**Figure 8:**
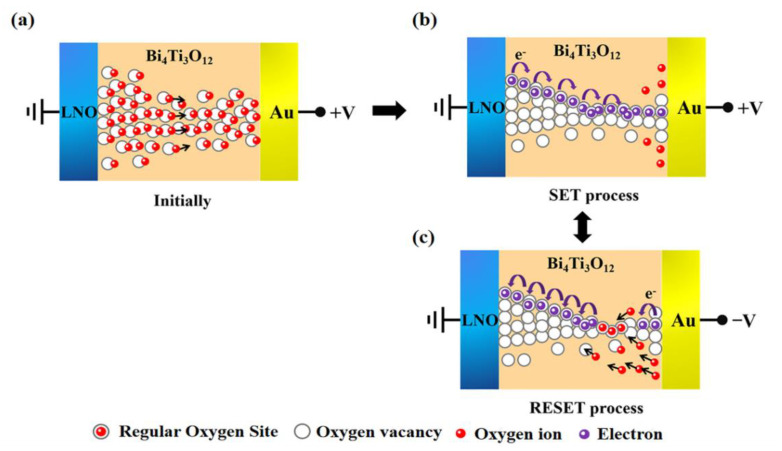
Model of an oxygen-vacancy-related filament. (**a**) Resistive switching at the initial state. (**b**) Set process of the device. (**c**) Reset process of the device.

According to the above analysis, it can be concluded that the RS characteristic is associated with the presence of numerous oxygen vacancies in the film, and the formation and fracture of conductive filaments composed of oxygen vacancies can lead to the transition between HRS and LRS [53,54]. As displayed in Figure 8, a typical conductive filaments theory was proposed to describe the effects of oxygen vacancies. The hollow circles represent oxygen vacancies in the BIT thin films, the red balls represent oxygen ions, and the purple pellets are the electrons in the film. Initially, the regular oxygen sites were evenly distributed in the film, as shown in Figure 8a, corresponding to the HRS of the device. An electrochemical reaction occurs when a forward bias is applied to the top Au electrode of the device. This electrochemical reaction can be written in Krὅger-vink notations as [55]:

(3) $$O_{o}\to V_{o}^{++}+O^{-2}$$

where $O_{o}$ represents an oxygen ion on a regular site, $V_{o}^{++}$ denotes oxygen vacancies with a double positive charge, and *O*^−2^ represents oxygen ions. The electrochemical reaction separates oxygen ions from the regular oxygen sites, and these oxygen ions can drift under the applied electric field, which is equivalent to the movement of the oxygen vacancy [56]. With the elevation in applied voltage, the injected electrons fill the defects and control the flow of electricity. The conduction mechanism of this process is controlled by SCLC. Due to the positive voltage acting on the Au electrode, oxygen ions drift toward the Au electrode and leave oxygen vacancies behind. At this point, the electrons are directed toward the Au electrode to form the conductive filament. As shown in Figure 8b, when the conductive filaments connected the LNO and Au electrode, a steep current simultaneously increased, and the SET process could be achieved [44]. When negative voltage was applied to the Au electrode, the opposite electric field forced the oxygen ions to move toward the LNO. The oxygen ion recombined with the oxygen vacancy, leading to the disruption of conductive filaments, as illustrated in Figure 8c. The device switches from HRS corresponding to the RESET process. The formation and rupture of the conductive filaments illustrated the carrier transport characteristics of the device, showing consistency with the RS performance, as well as the conduction mechanism of the device.

### 3.4. Conclusions

In summary, the Au/BIT/LNO/Si devices were prepared via the sol-gel process. First, the V-I characteristics of the devices annealed in air were investigated, and the results showed a high stability and reliability of bipolar RS properties. The ratio of R_HRS_/R_LRS_ of the device annealed in air reached about 10^2^, and the RS characteristic showed no significant deterioration after 100 successive cyclic tests. However, the R_HRS_/R_LRS_ ratio of the device annealed in O_2_ was only 10^1^, which is significantly lower than the device annealed in air. Through XPS test analysis of both devices, we found this was due to the effect of the concentration of oxygen vacancies in the films. We found that the oxygen vacancies have an enhanced effect on the resistance switching characteristics of BIT films. The conduction mechanism of the RS properties was also studied, and the results indicated that the conduction mechanism in HRS was regulated by SCLC, and the conduction mechanism of LRS was governed by Ohm’s law. Moreover, the formation and rupture of oxygen vacancy filaments were presented to specify the switching of the LRS and HRS states. In this study, the excellent bipolar RS properties of Au/BIT/LNO/Si devices were first reported. It is suggested that the BIT thin films are worthy of further research to extend the application fields of ferroelectric films in RRAM.

## Figures and Tables

**Table 1:** Comparison of resistive switching performance of RRAM devices.

Device Structure	ON/OFF	Endurance Cycles	Retention Time	Year/Ref.
Ag/BaTiO_3_/Nb:SrTiO_3_	~200	40	10^4^ s/85 °C	2020/[32]
SRO/BaTiO_3-__δ_/SRO	~7	270	—	2013/[33]
Au/BaTiO_3_/Nb:STO	~41	50	—	2017/[34]
Pt/BiFeO_3_/SRO	~750	—	10^3^ s/RT ^1^	2013/[35]
Al/BiFeO_3_/ITO	~2.3	10	—	2020/[36]
Ag/BiFeO_3_/FTO	~12	100	—	2020/[37]
Pt/Hf_0.5_Zr_0.5_O_2_/LSMO	~210	10^4^	10^3^ s/RT	2021/[38]
Cu/Ti/HfO_2_/TiN	~100	2000	10 years/200 °C	2019/[39]
Ag/HfO_2_/Pt	~10^5^	10^8^	>1 day/150 °C	2021/[40]
TiN/Sr:HfO_2_/Pt	~50	50	—	2018/[41]
Au/Bi_4_Ti_3_O_12_/LNO/Si	~100	100	10^4^ s/85 °C	This work

^1^ RT, measured at room temperature.

## Data Availability

The data presented in this study are available on request from the corresponding author.

## Supplementary Materials

The following are available online at https://www.mdpi.com/article/10.3390/nano11102705/s1, Figure S1: (a,b) XPS survey spectrum, (c,d) Bi 4f high resolution spectra and (e,f) Ti 2p high resolution spectra of air-annealed and oxygen-annealed BIT films respectively.
