# Supplementary material for: Excellent Bipolar Resistive Switching Characteristics of Bi4Ti3O12 Thin Films Prepared via Sol-Gel Process
Source: Nanomaterials (Basel). 2021 Oct 14;11(10):2705. doi: 10.3390/nano11102705 (PMC8537946; doi:10.3390/nano11102705)
Supplement: Supplementary file 1 [file nanomaterials-11-02705-s001.zip › nanomaterials-1390379-supplementary.pdf]

nanomaterials

MDPI

Supplementary Materials

Excellent bipolar resistive switching characteristics of $Bi_4Ti_3O_{12}$ thin films prepared via sol-gel process

He-Chun Zhou, Yan-Ping Jiang *, Xin-Gui Tang, Qiu-Xiang Liu, Wen-Hua Li and Zhen-Hua Tang

School of Physics and Optoelectric Engineering, Guangdong University of Technology; Guangzhou Higher Education Mega Centre, Guangzhou, 510006, P. R. China; hechunzz@163.com (H.-C. Z.); xgtang@gdut.edu.cn (X.-G. T.); liuqx@gdut.edu.cn (Q.-X. L.); liwenhuat@gdut.edu.cn (W.-H. L.); tangzh@gdut.edu.cn (Z.-H. T.)

* Correspondence: ypjiang@gdut.edu.cn

The chemical composition and elemental oxidation state of BIT films were investigated by XPS. The XPS model is Escalab 250Xi and the instrument manufacturer is Thermo Fisher (Manchester, England). The vacuum degree in the chamber is better than 5.0×10-10 mBar. During the analysis, the beam spot of the X-ray source (Al target) is 650 μm, and the voltage and current are 15 kV and 15 mA, respectively. This information is also added to the supporting material in the manuscript. The spectral line fitting method we use is Gaussian line shape, and the type of background subtraction is Shirley background subtraction. Figure S1(a) and (b) shows the XPS survey spectrum of the BIT films, confirming the presence of major elements such as Bi 4f, Ti 2P, O 1s and C 1s, where the C 1s peak is derived from the carbon adsorbed on the sample surface. The XPS spectra of the samples were fitted to analyze the precise positions and valence states of the Bi 4f, and Ti 2p. As shown in Figure S1(c) and (d), the Bi 4f high resolution spectra was deconvoluted into two clearly separated Gaussian peaks at 159.2 and 164.5 eV, which corresponded to the Bi 4f7/2 and Bi 4f5/2, respectively, indicating the presence of Bi^{3+}. The Ti 2p high resolution spectra (Figure S(e) and (f)) presents a broad bump at within 465 eV, contributing to the partial overlapping of Bi 4d3/2 and Ti 2p1/2. After the peak fitting analysis, the peaks of Ti 2p3/2 and Ti 2p1/2 could be achieved at 458.2 and 464.1 eV, respectively, proving the presence of Ti^{4+}.

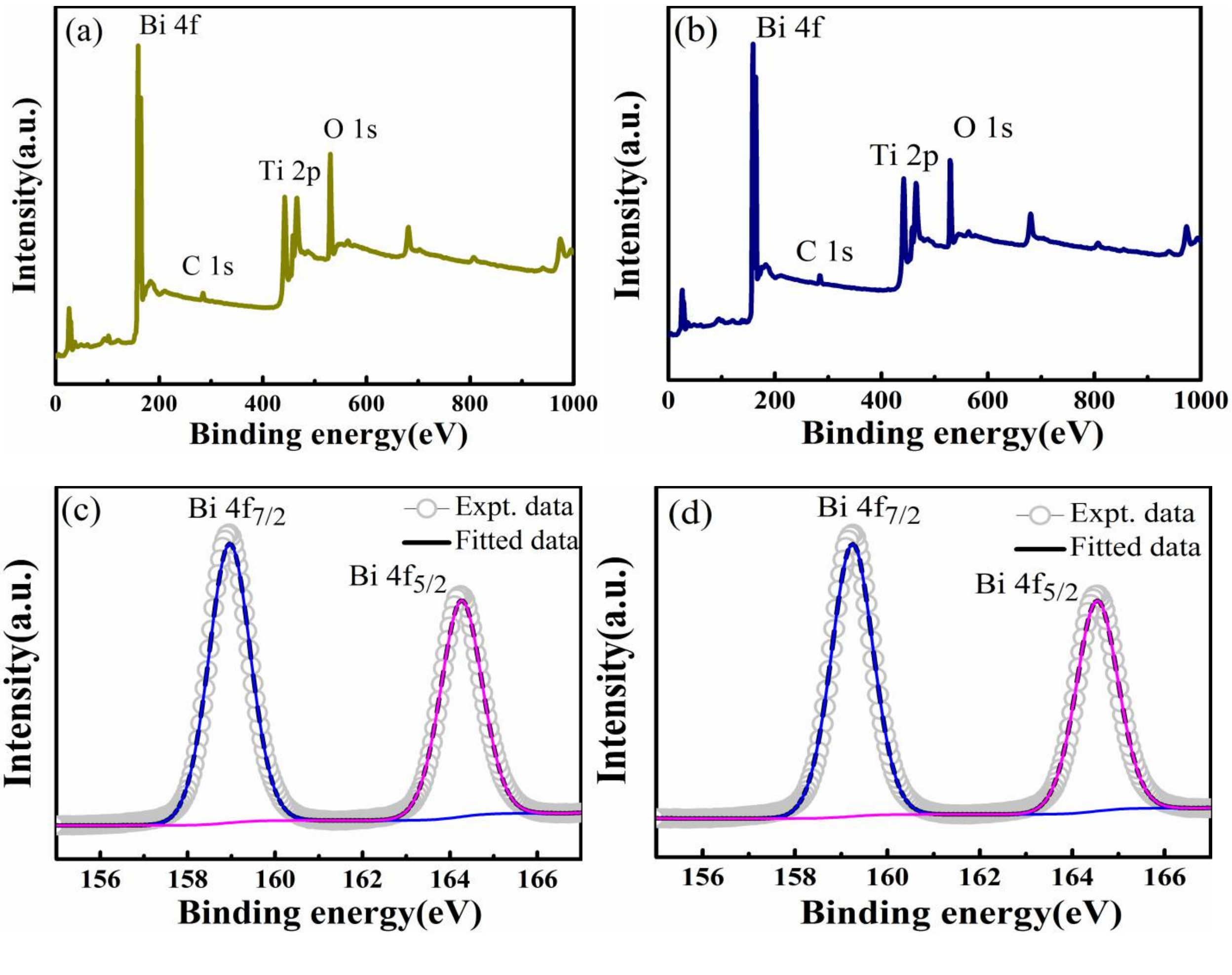

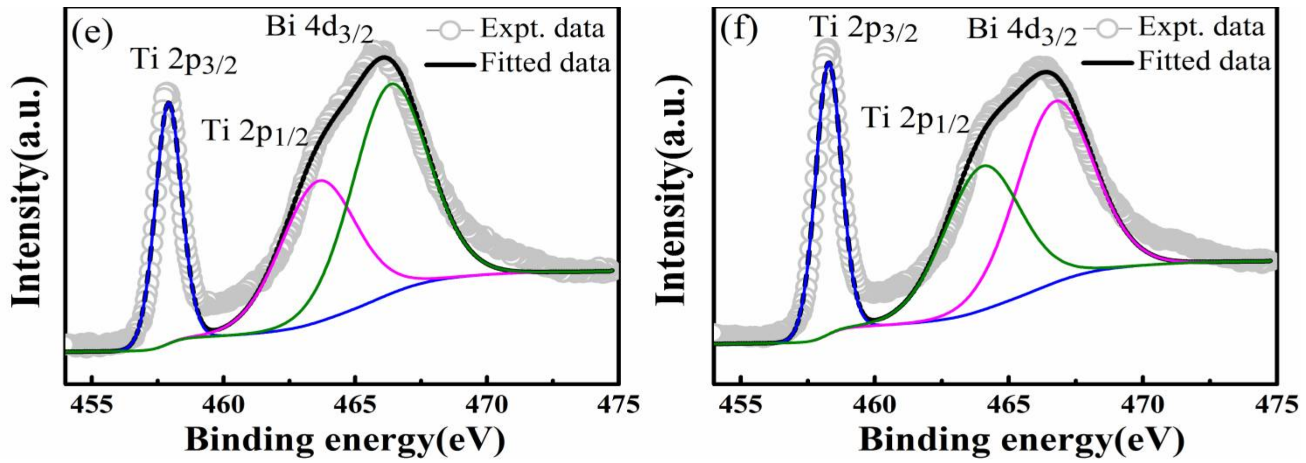

Figure S1. (**a**-**b**) XPS survey spectrum, (**c**-**d**) Bi 4f high resolution spectra and (**e**-**f**) Ti 2p high resolution spectra of air-annealed and oxygen-annealed BIT films respectively.
